# Impact of soil application with selenite and selenate on ‘soil-highland barley-dietary’ system in Tibet

**DOI:** 10.3389/fpls.2025.1589810

**Published:** 2025-06-18

**Authors:** Chenni Zhou, Fei Duan, Jiangke Wang

**Affiliations:** ^1^ Research Center of Agricultural Economy, College of Economics, Sichuan University of Science and Engineering, Yibin, China; ^2^ Key Laboratory of Alpine Vegetation Ecological Security in Tibet, Institute of Tibet Plateau Ecology, Tibet Agricultural and Animal Husbandry University, Nyingchi, China

**Keywords:** highland barley, Se spices, soil Se fraction, Se intake, biofortification

## Abstract

**Introduction:**

Selenium (Se)-fortified foods have demonstrated efficacy in augmenting dietary Se intake and ameliorating human Se nutritional status. To mitigate Se deficiency-related health risks in Tibetan populations, systematic biofortification trials targeting highland barley, the primary staple crop in Tibet, are imperative.

**Methods:**

Highland barley was subjected to soil-applied selenate (SeVI) and selenite (SeIV) at seven rates (0, 5, 15, 25, 50, 75, and 100 g·ha^-1^), followed by comprehensive evaluation of Se transfer dynamics within the soil-crop-diet continuum across Tibet’s agricultural regions.

**Results and discussion:**

Exogenous Se application significantly increased the Se content in highland barley grains (*p* < 0.05), with SeVI proving to be more effective than SeIV. Selenomethionine (SeMet) accounted for 78-85% of total Se species in grains, and SeIV applications yielding 1.7 times higher organic Se conversion rates compared to SeVI treatments. Se application not only elevated the total Se concentration but also concurrently increased the bioavailable Se fractions in the soil, thereby enhancing Se translocation within the plant. Dietary exposure assessment indicated that application rates of 75 g·ha^-1^ SeIV and 50 g·ha^-1^SeVI were optimal, as they satisfied the recommended daily intake (55 μg·day^-1^) for Tibetan adults while keeping soil Se below 3.0 mg·kg^-1^. This study demonstrated that soil application of 75 g·ha^-1^ SeIV or 50 g·ha^-1^ SeIV achieved effective biofortification without ecological risk, providing a sustainable solution for mitigating Se deficiency in Tibetan agroecosystems.

## 1 Introduction

As an indispensable micronutrient, selenium (Se) plays pivotal biochemical roles through its incorporation into selenoproteins, including glutathione peroxidase (GPx), iodothyronine deiodinases (DIO), and thioredoxin reductase (TXNRD) systems, which mediate redox homeostasis and thyroid hormone metabolism (Wang et al., 2020b). Epidemiological evidence has established Se deficiency as a risk factor for >40 human pathologies, including Keshan cardiomyopathy and Kashin-Beck osteoarthropathy (Navarro-Alarcon and Cabrera-Vique, 2008). Dietary biofortification through Se supplementation, while preserving cultural dietary patterns, represents the most viable approach to achieve optimal serum Se concentrations (58~150 μg·L^-1^) across populations, as demonstrated by longitudinal cohort studies (Lemire et al., 2011; Krishnakumar et al., 2018).

Soil application of exogenous Se is an agronomy practice aimed at enhancing both the total and bioavailable Se content in the soil, thereby increasing the Se concentration in the edible parts of crops (Broadley et al., 2010).The Finnish National Se Program (1984~2001) pioneered large-scale Se fertilization, establishing the first government-mandated Se agronomic fortification protocol. Systematic SeIV fertilization increased population-level dietary Se intake from 0.04 mg·day^-1^·10 MJ^-1^ in 1985 to 0.08 mg·day^-1^·10 MJ^-1^ in 2001, corresponding with elevated plasma Se concentrations (0.89~1.4 µmol·L^-1^, *p* < 0.001) (Alfthan et al., 2015). Se bioavailability demonstrates species-specific responses modulated by: crop Se assimilation genetics, Se speciation and application rates. In maize (*Zea mays* L.) cultivation trials on Loess Plateau in China, SeVI application (1 kg·ha^-1^) enhanced grain Se content from 0.12 ± 0.03 to 0.33 ± 0.05 µg·kg^-1^ dry weight (DW) across two phenological cycles (Wang et al., 2013a). Optimal Se dosage (50 g·ha^-1^) maximized rice yield outputs (8163.5 kg·ha^-1^; +11.5%), while supra-optimal applications (>75 g·ha^-1^) induced yield penalties through selenocysteine misincorporation in proteins (Zhang et al., 2014). Contrastingly, buckwheat (*Fagopyrum esculentum Moench*) exhibited 2.3-fold Se accumulation in seeds under selenite treatment (25 g·ha^-1^), yet demonstrated phytotoxicity symptoms at application rates exceeding 50 g·ha^-1^ (Jiang et al., 2015). A potential explanation for these observations is that Se application can alter the transformation and proportions of Se fractions in the soil. Controlled pot experiments demonstrated Se supplementation (10~100 mg·kg^-1^) increased bioavailable fractions: water-soluble Se (SOL-Se, +120%), exchangeable Se (EXC-Se, +85%), while reducing residual forms (RES-Se, -40%) through enhanced microbial selenate reductase activity (Ali et al., 2017). Soil Se application has been widely utilized to enhance Se concentrations in staple crops like rice (Zhang et al., 2014; Wang et al., 2013b) and wheat (Ali et al., 2017), there remains a notable gap in the research on Se biofortification across different crops in Se-deficient regions.

An estimated 1.1 billion people globally inhabit Se-deficient regions (soil Se content < 0.125 mg·kg^-1^), predisposing populations to compromised selenoprotein biosynthesis (Paikaray, 2016; Jones et al., 2017). 68% of county-level administrative units was in Se-deficient zones (0.08~0.15 mg·kg^-1^), where 53% of adults exhibit suboptimal dietary Se intake associated with elevated hypothyroidism risk in China (Dinh et al., 2018). The Tibetan Autonomous Region demonstrates particularly high Se-deficiency comorbidities, with Kashin-Beck disease (KBD) prevalence reaching 12.8% in agricultural counties versus 2.1% nationally, and Keshan cardiomyopathy incidence 6-fold higher than lowland regions (Winkel et al., 2012; Zhang et al., 2018). While government interventions (2006~2015) including Se-fortified salt distribution (coverage: 58% target population) temporarily reduced KBD incidence by 37%, cost-benefit analysis revealed unsustainable implementation costs and logistical barriers in remote highland villages (elevation: > 3800 m ASL) (Li et al., 2012, 2003; Yu et al., 2019). Highland barley (*Hordeum vulgare* L. var. *nudum* Hook. f.) dominates Tibetan agriculture, occupying 65000 km^2^ (82% of arable land) at elevations 3500~4500 m, providing 72% of daily caloric intake and 58% of dietary protein for rural communities (Guo et al., 2017). Our previous research has shown that highland barley is the primary source of dietary Se for rural Tibetan residents, contributing 34.2% of their Se intake (Zhou et al., 2022). Meanwhile, pre-biofortification analyses revealed inadequate Se contribution of highland barley (10.3% of RDA) with mean grain concentrations 0.08 mg·kg^-1^, resulting in 43 μg·day^-1^ intake deficit (Zhou et al., 2022). Given the wide-spread consumption of highland barley in Tibet, the production of Se-enriched highland barley grain could be a promising strategy to improve dietary Se intake among Tibetan populations.

Overall, this study systematically addresses three objectives: (i) quantify Se allocation patterns in highland barley tissues under SeVI and SeIV fertilization; (ii) evaluate Se fractionation dynamics in rhizosphere soils; and (iii) establish safe and optimal Se application thresholds through dietary exposure assessment.

## 2 Materials and methods

### 2.1 Overview of the experimental site

**Figure 1:**
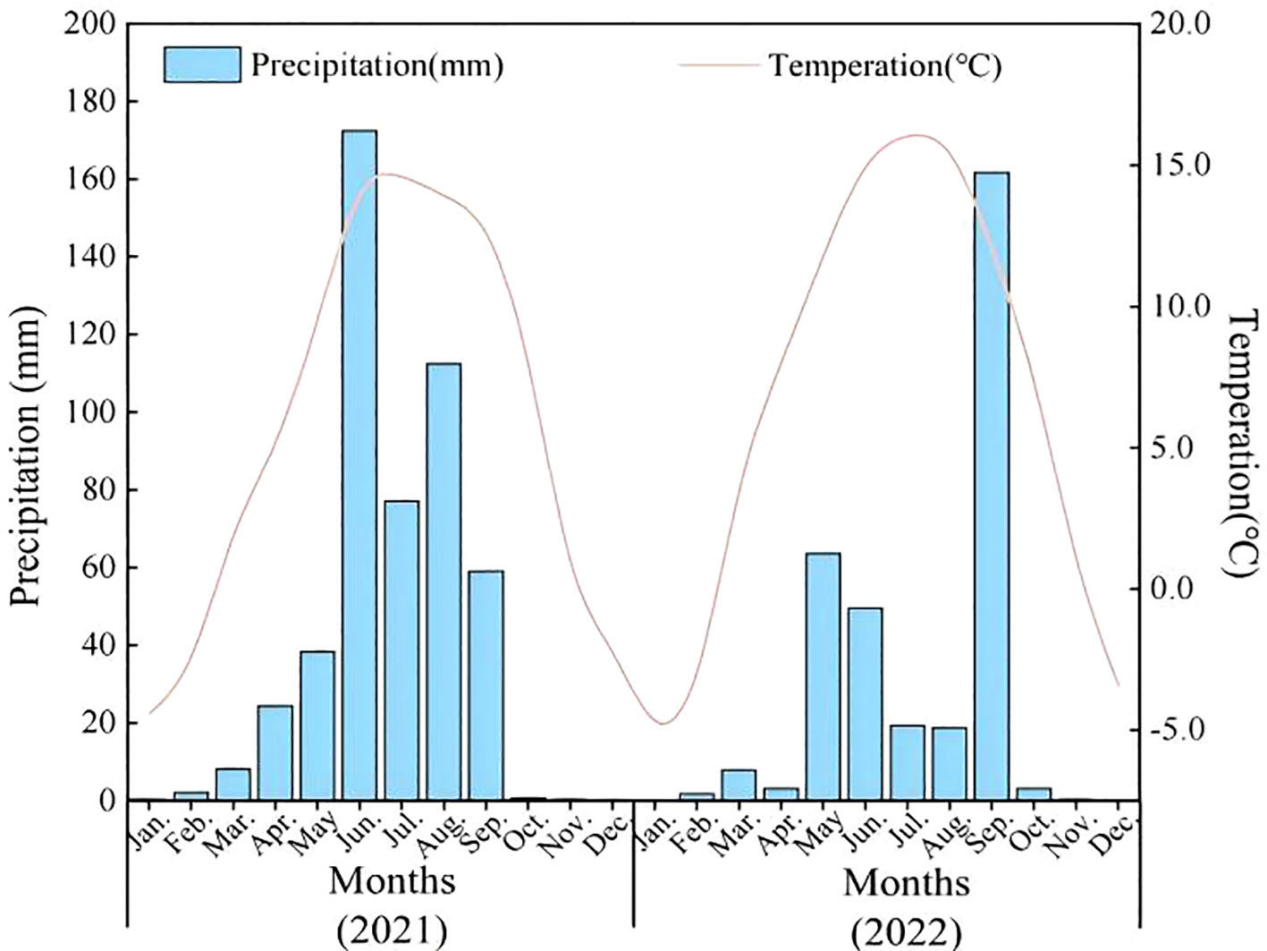
Mean monthly temperature (°C) and mean monthly precipitation (mm) during the experiment.

Field trials were implemented during consecutive growing seasons (2021~2022) at 29°53’N 91°07’E in Bairang Village, Linzhou County, Tibet Autonomous Region (elevation: 3890 m ASL), representing a typical high-altitude agricultural system. The site exhibits a typical plateau monsoon climate with mean annual precipitation of 491 mm (80% occurring June-September), mean temperature of 5.2 °C, and diurnal thermal amplitude exceeding 15 °C. Annual solar radiation reaches 7892 MJ·m^-2^, with 3000 sunshine hours and 122 frost-free days annually. USDA classified the soil as sandy clay loam (clay: 15%, sand: 64%, silt: 21%) with pH 7.2. Key properties included: organic matter content 20.08 g·kg^-1^, total N content 1.16 g·kg^-1^, total P content 0.84 g·kg^-1^, total S 0.28 g·kg^-1^, total Se content 0.05 mg·kg^-1^, CaCO_3_ content 18.79 g·kg^-1^, CEC 7 cmol(+)/kg; Fe 8.4 mg·kg^-1^; Mn 9.5 mg·kg^-1^; Zn 0.66 mg·kg^-1^; Cu 0.78 mg·kg^-1^; B 0.47 mg·kg^-1^. Total soil Se content (0.05 ± 0.01 mg·kg^-1^, <0.125 mg·kg^-1^) classified the site as Se-deficient per the Chinese Soil Se Threshold System (Tan et al., 2002). The meteorological conditions of the experimental site from 2021 to 2022 were shown in **Figure 1**.

### 2.2 Field trial setup

The highland barley (*Hordeum vulgare* L. var. *nudum* Hook. f.) cultivar ‘Zangqing 2000’ was cultivated during the 2021–2022 growing seasons (sowing: 18 Apr 2021/25 Apr 2022; harvesting: 28 Aug 2021/5 Sep 2022) under standard agronomic practices. Sodium selenite (Na_2_SeO_3_, CAS 10102-18-8, SeIV) and sodium selenate (Na_2_SeO_4_, CAS 13410-01-0, SeVI) were procured from Sigma-Aldrich (Sigma-Aldrich, St. Louis, MO, USA). A complete randomized block design was implemented with seven Se rates (0, 5, 15, 25, 50, 75, 100 g·ha^-1^) applied as SeIV or SeVI, generating 14 experimental treatments (n=56 plots). Treatments were randomly allocated to experimental units (4 m×5 m plots) in quadruplicate using R v4.2.1 randomization algorithms. Individual plots were separated by 1 m wide buffer zones filled with inert quartz sand to prevent cross-contamination, maintaining a 2:1 plot-to-buffer area ratio. Based on the specified Se application rates and plot size, the required amounts of SeIV or SeVI were carefully calculated and weighed using an analytical balance. The compounds were then fully dissolved in 2 liters of deionized water to prepare a homogeneous solution, which was uniformly applied to the soil surface of each plot using a manual sprayer after sowing. Standard cultivation and management practices were followed throughout the experiment.

### 2.3 Sample collection and preparation

At the maturity stage, highland barley plants within a 1 m^2^ area in the center of each experimental plot were collected, along with corresponding soil samples from the 0–20 cm layer. The plant samples were thoroughly washed with distilled water and subsequently divided into three parts: roots, straws, and grains. After surface moisture was allowed to drain, the samples were placed in an oven at 65 °C and dried to a constant weight, which generally required approximately 72 hours. The dry weight of each part was meticulously recorded. The dried plant samples were then ground into a fine powder using a stainless steel pulverizer and sieved through a 0.5 mm nylon mesh for further analysis. The soil samples were naturally air-dried, and following the removal of impurities such as plant residues and gravel, they were sieved in preparation for subsequent analysis.

### 2.4 Chemical analysis

#### 2.4.1 Determination of Se concentration in soil and highland barley plants

0.250~0.251 g of plant samples were digested with 8 mL of HNO_3_. The procedure was as follows: 80 °C for 1.5 h, 120 °C for 1.5 h, 150 °C for 3 h, and 160 °C for 0.5 ~ 1.5 h. Soil samples were digested with a 3:2 (v/v) mixture of HNO_3_ and HClO_4_, and the remaining acid digestion steps were the same as those for plant samples. The concentration of Se in the digested solution were measured by Inductively coupled plasma mass spectrometry (ICP-MS, iCAP Q, Thermo Scientic USA.). A wheat grain standard sample (GBW08503c, Academy of State Administration of Grain, SAAG) and a blank were simultaneously digested with the test samples for the QA/QC protocol. The average recovery rate of Se was 85% ~ 105% (Wang et al., 2021).

#### 2.4.2 Determination of Se speciation in highland barley grain

0.40 g of barley grain samples was hydrolyzed by adding 5 mL of 8 mg·mL^-1^ protease XIV (Sigma Chemical Co., St. Louis, MO, USA). The mixture was shaken at 125 rpm for 24 h at 37 °C. Following centrifugation at 12000 rpm for 15 min, the mixture was filtered through a 0.22 µm cellulose nitrate filters and analyzed using high performance liquid chromatography-ultraviolet treatment-hydride generation-atomic fluorescence spectrometry (HPLC-UV-HG-AFS). Then, Se speciation in barley grain was analyzed by the HG-AFS detection system. The HPLC system was equipped with a Hamilton PRP-X100 (internal diameter=4.1 mm, length=250 mm, particle size=10 µm; Hamilton, Switzerland). The Se standards were Se-methyl-selenocysteine (MeSeCys) (Sigma-Aldrich, St. Louis, MO, USA), selenocystine (SeCys2) (Sigma-Aldrich, St. Louis, MO, USA), seleno-methionine (SeMet) (Sigma-Aldrich, St. Louis, MI, USA), selenite (SeIV), and selenate (SeVI). To evaluate the effect of protease XIV enzymatic hydrolysis on the extraction of Se speciation in barley grain, the total Se concentration in the supernatant was determined after centrifugation (Wang et al., 2020a). The extraction rate was the ratio of the total Se in the supernatant to the total Se in sample.

#### 2.4.3 Sequential extraction of soil Se fractions

(i) Extraction of soluble Se (SOL-Se): 10 mL 0.25 M KCl was added to 1.0 g soil in a 100 mL polypropylene centrifuge tube. Then, the tube was shaken at 200 rpm at 25°C for 1 h. The mixture was centrifuged for 10 min at 4000 rpm, and filtered through a 0.45 μm filter. The remaining precipitate was used for the next step of extraction. The same centrifugation and filtration steps were conducted after each of the following extraction procedure. (ii) Extraction of exchangeable and carbonate-bound Se (EXC-Se): 10 mL 0.7 M KH_2_PO_4_ (pH = 5.0) was added to the tubes and shaken at 200 rpm for 4 h at 25°C. (iii) Extraction of Se bound to iron and manganese oxides (FMO-Se): 10 mL 2.5 M HCl was added to the remaining soil. The capped vials were then heated in a water bath at 90°C for 50 min. The centrifuge vials were also shaken intermittently. (iv) Extraction of organic matter bound Se (OM-Se): 8 mL 5% K_2_S_2_O_8_ and 2 mL concentrated HNO_3_ were added to each remaining soil sample, and the vial was heated for 3 h in a water bath at 95°C. The tube was intermittently shaken from time to time. (v) Extraction of residual Se (RES-Se): The soil residuals were transferred into a Teflon crucible with 8 mL concentrated HNO_3_ and 2 mL concentrated HClO_4_. The crucibles were covered and heated at 170°C in a sand bath until the soil appeared white or gray. After acid digestion, the solution was transferred to a 25 mL volumetric flask with deionized water (Wang et al., 2012).

### 2.5 Data analysis

#### 2.5.1 Parameters characterizing Se behavior in soil

Soil Se redistribution index (*U_ts_*), relative binding intensity of Se with soil (*I_R_*), and the mobility factor (*MF*) were used to represent the impact of Se application on soil Se and Se fractions (Ali et al., 2017). The calculation formula of *U_ts_*, *I_R_*, *MF* were as follows:


$$U_{ts}=\sum_{i=1}^{5}(F_{i}\times \frac{F_{i}}{F_{ci}})$$

$$I_{R}=\sum_{i=1}^{5}(F_{i}\times i^{2})/5^{2}$$

$$MF=\left(F_{1}+F_{2}\right)/\sum_{i=1}^{5}F_{i}$$

In which, *F_i_* represents the proportion of a specific Se fraction in the Se-treated soil, *F_ci_* represents the proportion of a specific Se component in the control soil, *i* represents the extraction step, *F_1_* represents the proportion of SOL-Se in the soil, and *F_2_* represents the proportion of EXC-Se in the soil.

#### 2.5.2 Estimated dietary Se intake analysis

The estimate of dietary Se intake (*DI*, µg·adult^-1^·day^-1^) was calculated according to this equation: *DI*=*C_highland barley_* × *W_highland barley_*, where *DI* (µg·adult^-1^·day^-1^) is daily Se intake estimation per adult, *C_highland barley_* (µg·g^-1^) is the Se concentration in highland barley grain in this study, *W_highland barley_* (g·day^-1^·adult^-1^)is the mean daily consumption of highland barley per adult (0.4 kg·adult^-1^·day^-1^) (Tashi et al., 2013; Di et al., 2023).

#### 2.5.3 Statistical analysis

All experimental data were expressed as the mean ± standard deviation (mean ± SD) from four replicates. The effects of exogenous Se types, Se application rates, and years on Se concentration and content in different parts of highland barley (grain, straw, and root) were analyzed using a multi-factor analysis of variance (ANOVA). When significant differences were found (*P* < 0.05), pairwise comparisons among different Se application treatments were performed using Tukey’s LSD test. Statistical analyses were conducted using SPSS version 23.0 (IBM Corp., Armonk, NY, USA), with a significance level of *P*<0.05. Graphs were generated using Origin 2024b (OriginLab, Northampton, MA, USA) and R version 4.2.3 (R Development Core Team, 2023).

## 3 Results

### 3.1 Se concentration and transformation in highland barley

**Figure 2:**
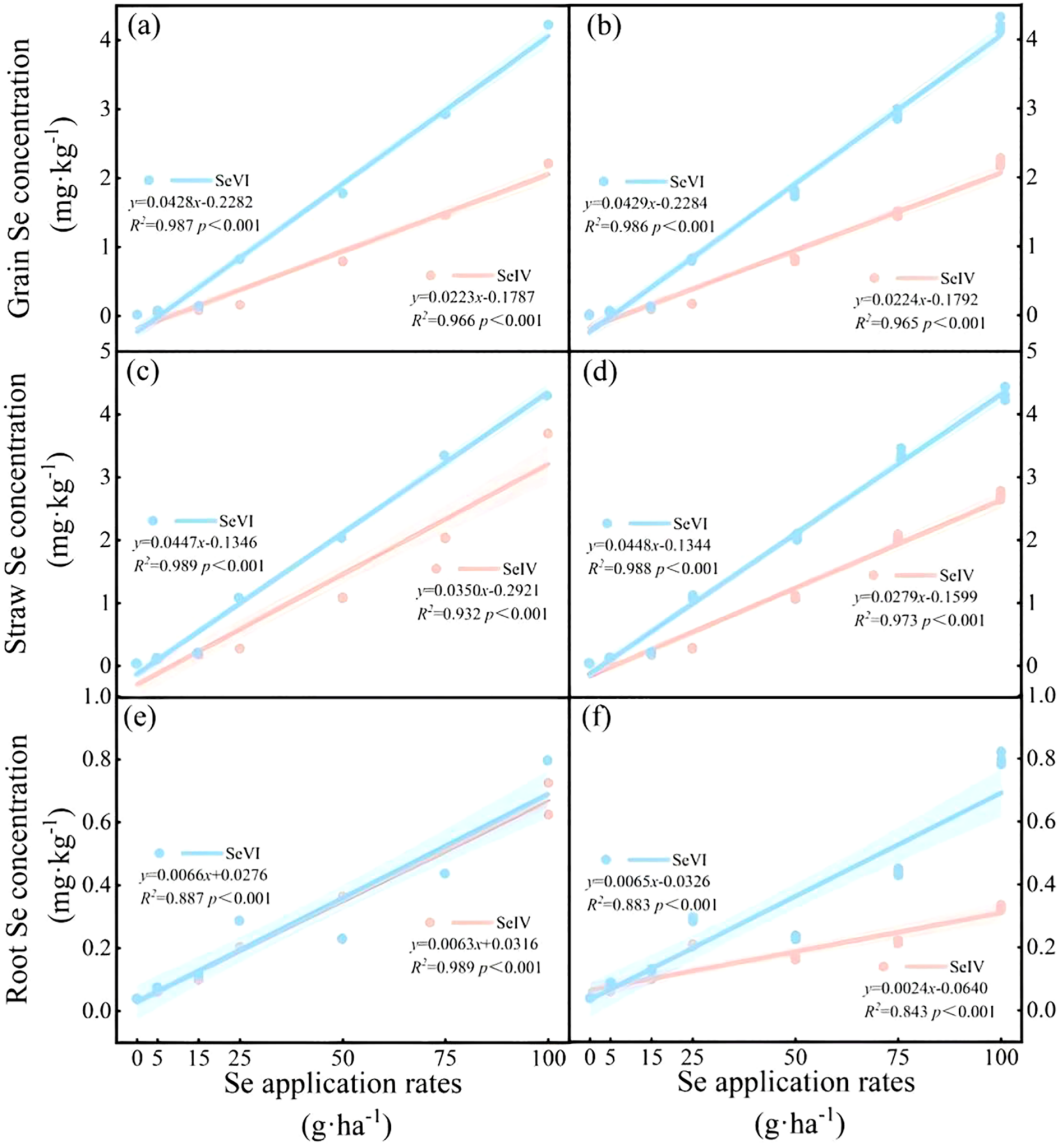
Relationships between total Se concentrations in grains, straws and roots of highland barley and Se application rates (**a, c, e** ~ Year 2021; **b, d, f** ~ Year 2022).

Soil application of exogenous Se significantly elevated Se concentrations in all barley tissues (*p* < 0.01), with both SeIV and SeVI treatments showing dose-dependent accumulation patterns (**Supplementary Figure S1**, **Figure 2**). Dose-response analysis revealed significant linear correlations between application rates and tissue Se accumulation (R^2^ = 0.83~0.94, *p* < 0.001), particularly evident in grains (**Figure 2**). In the 2021 trial, SeIV application (5~100 g·ha^-1^) induced 2.44~122.73-fold, 2.75~71.02-fold, and 1.57~8.34-fold increases in grain, straw, and root Se concentrations, respectively, compared to non-treated controls (**Supplementary Figure S1**). The 2022 trial demonstrated greater bioaccumulation with SeVI, showing 4.43~235.27-fold increases in grains, 3.35~113.09-fold increases in straw, and 1.57~20.66-fold increases in roots (**Supplementary Figure S1**). Obviously, the overall efficacy of soil-applied SeVI was superior to SeIV. Optimal differentiation occurred at moderate application levels (25 g·ha^-1^), where SeVI produced 4.98-fold and 3.87-fold greater Se accumulation in grains and straw compared to SeIV (*p* < 0.01; **Supplementary Figure S1**, **Figure 2**). At maximum application (100 g·ha^-1^), root Se concentrations under SeVI treatment remained 2.45 fold higher than SeIV equivalents, suggesting partial rhizospheric conversion of SeVI to immobile forms (*p* < 0.01; **Supplementary Figure S1**, **Figure 2**).

**Figure 3:**
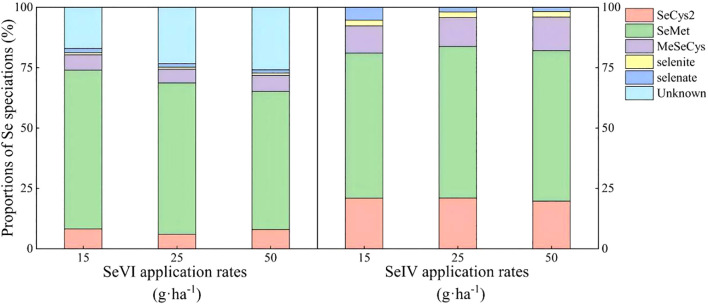
Se speciation distribution in highland barley grains under soil application of exogenous Se (2021).

Organic Se constituted 78~85% of total Se species in highland barley grains across treatments (**Figure 3**). A dose-dependent decline in organic Se proportion (80.35%→71.84%) and a speciation hierarchy (SeMet > SeCys2 > MeSeCys > selenite) was observed under SeVI treatments with corresponding SeMet reduction (65.77%→57.14%). An increasing trends of total organic Se (92.33%→95.98%) and SeMet proportion (60.09%→62.22%) under SeIV application was also observed. The application of SeIV produced a 1.2-fold higher SeCys2 accumulation under SeIV versus SeVI treatment (*p* < 0.01), while no significant difference was observed in the proportion of MeSeCys between two treatments (*p* < 0.05).

### 3.2 Total Se concentration and Se fractions in soil

**Figure 4:**
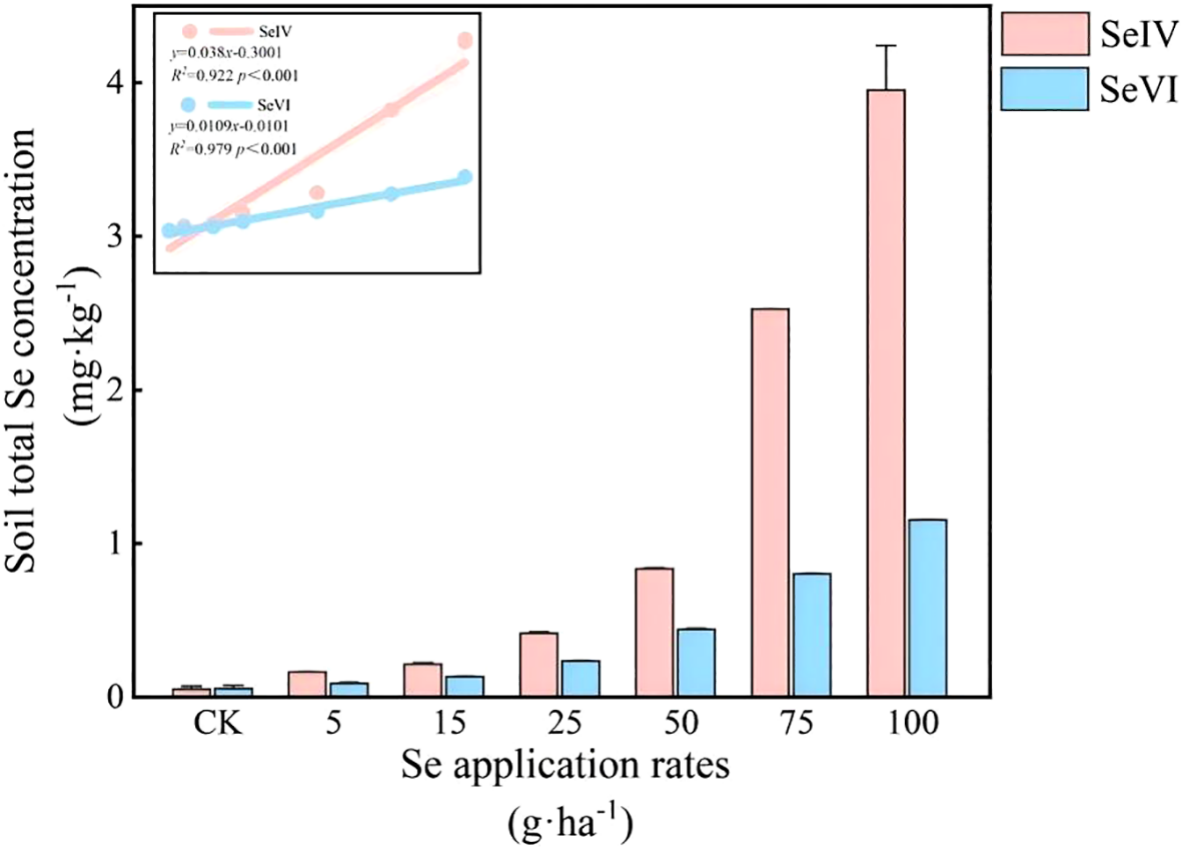
Effects of exogenous Se application on total Se concentration in soil.

SeIV and SeVI soil amendments (5~100 g·ha^-1^) increased total soil Se (*STSe*) by factors of 3.16~76.80 and 1.65~15.47 respectively relative to control plots (*p* < 0.001), demonstrating dose-dependent accumulation patterns (**Figure 4**). Linear regression analysis revealed strong positive correlations between application rates and *STSe* for both exogenous Se species (R^2^ = 0.96~0.98, *p* < 0.001). SeIV-amended soils retained 42~68% more *STSe* than SeVI-treated counterparts at equivalent application rates (*p* < 0.001), indicating stronger soil binding capacity of SeIV (**Figure 4**). Exogenous Se application enhanced bioavailable fractions (SOL+EXC-Se) from 26.07% in controls to 37.03~65.45% (*p* < 0.001), with SeVI showing 12~18% greater bioavailability than SeIV at equivalent doses.

**Figure 5:**
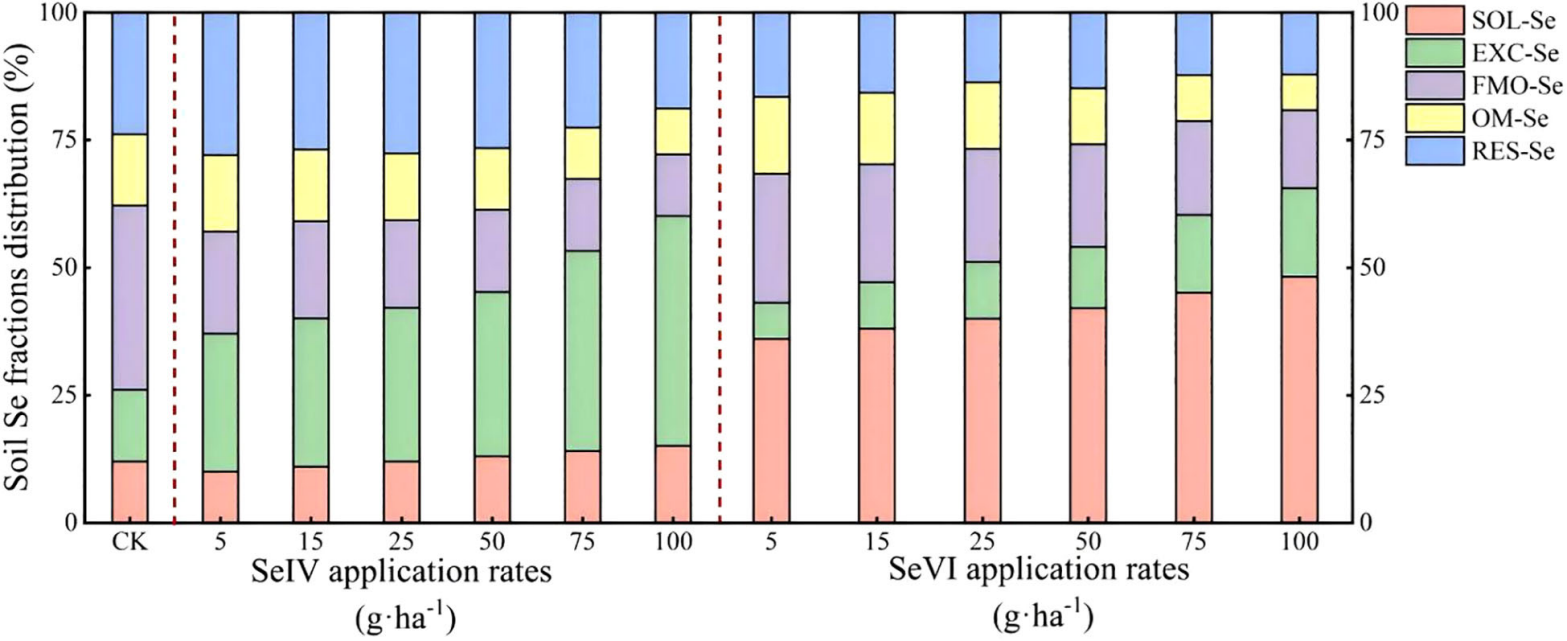
Effects of exogenous Se application on soil Se fractions.

SeIV application induced a marked reduction in FMO-Se proportions (36.06% to 15.08%) concurrent with EXC-Se increases from 27.01% to 45.05% (*p* < 0.01). The resultant speciation hierarchy under SeIV treatment established EXC-Se (45.05%) as the dominant fraction, followed by RES-Se (23.82%), FMO-Se (15.08%), OM-Se (14.04%), and SOL-Se (12.03%) (**Figure 5**). Following SeVI application, the proportion of SOL-Se markedly increased from 36.03% to 48.15%, wheras FMO-Se and OM-Se notably decreased with increasing Se application rates. Compared to the control (23.82%), SeVI application reduced the proportion of RES-Se from 16.59% to 11.62%. Following SeVI application, the distribution of Se fractions in the soil followed the order: SOL-Se > FMO-Se > RES-Se > OM-Se > EXC-Se (**Figure 5**).

### 3.3 Correlation analysis between Se concentrations of highland barley and parameters characterizing Se behavior in soil

**Figure 6:**
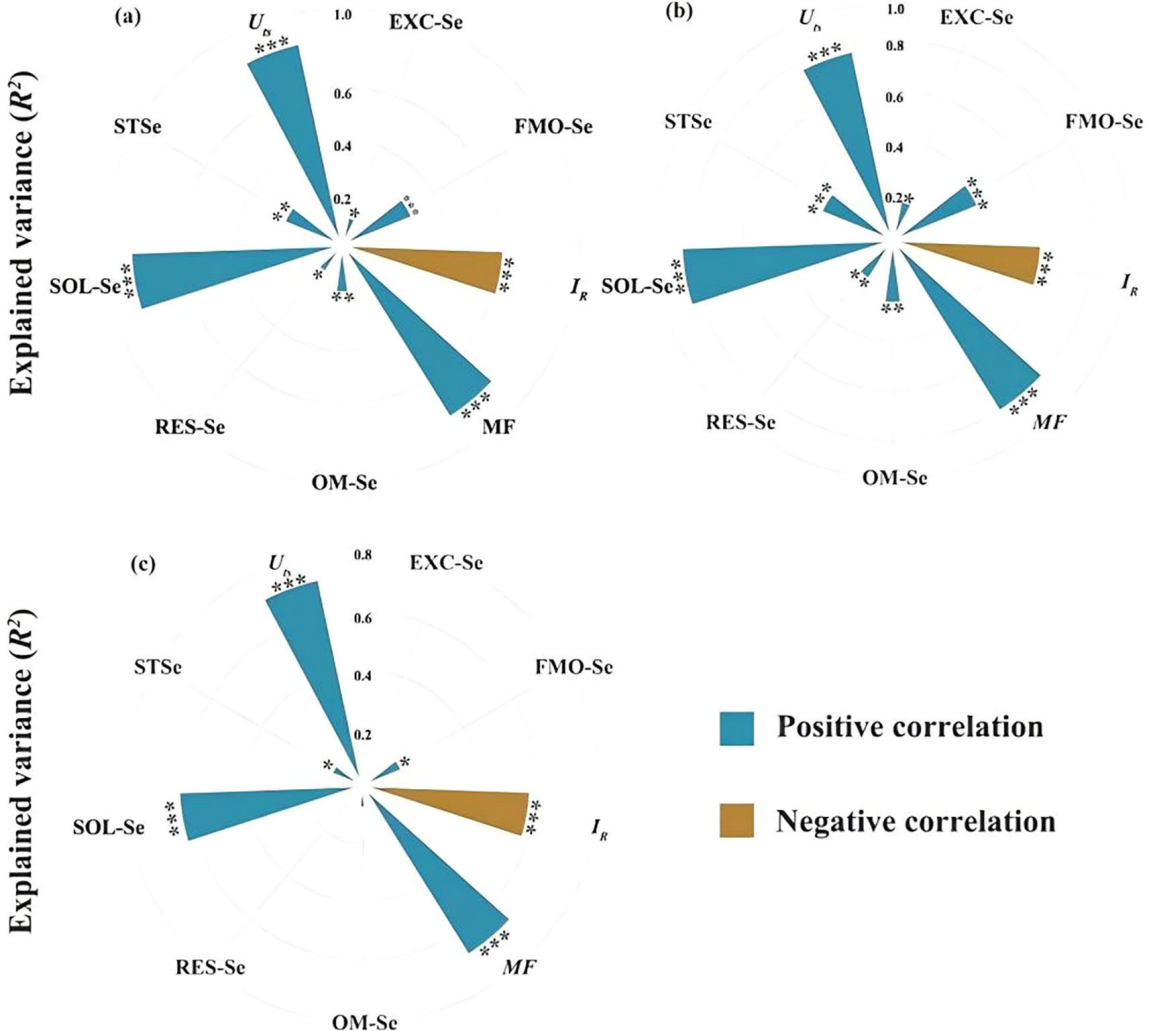
Relationships between Se concentrations in grains **(a)**, straws **(b)**, roots **(c)** of highland barley and total Se concentration in soil, Se fractions, Se redistribution index (*U_ts_*), relative binding intensity of Se with soil (*I_R_*), mobility factor (*MF*). Blue indicates positive correlation, while brown indicates negative correlation.*** indicates *p* < 0.001,** indicates *p* < 0.01; * indicates *p* < 0.05.

**Figure 7:**
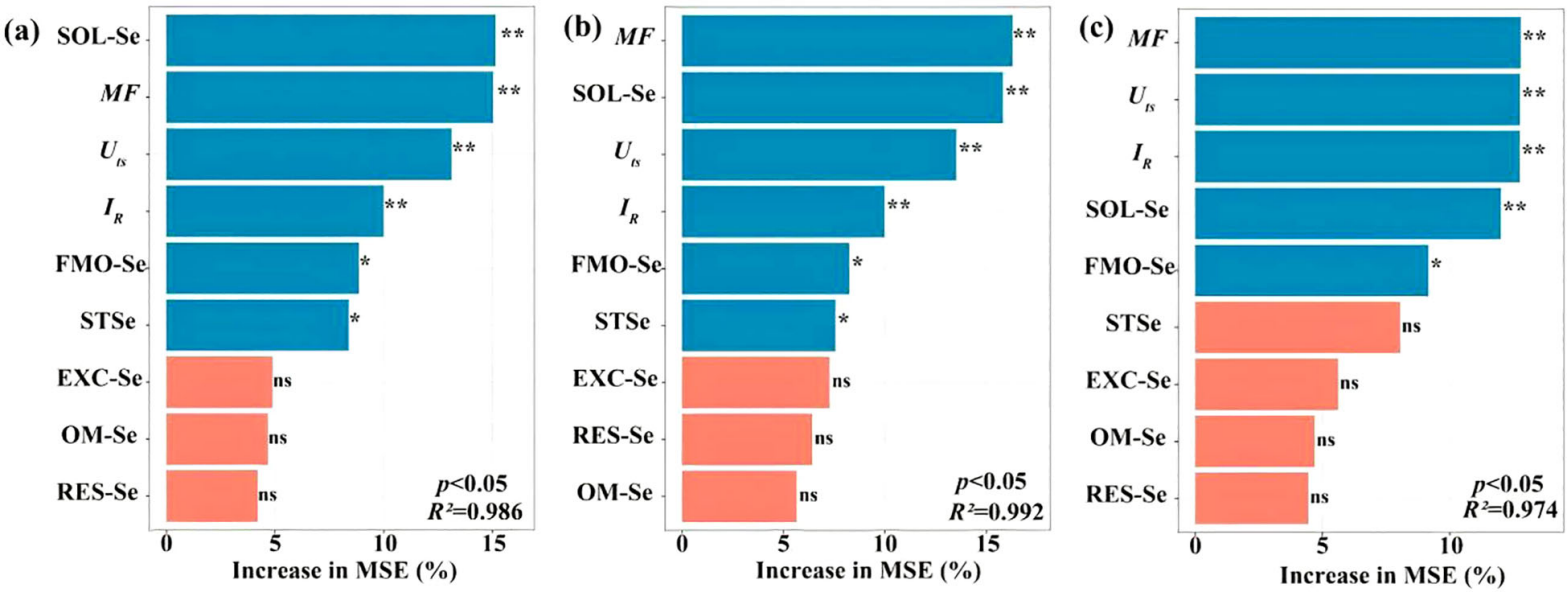
Relative importance of explanatory variables affecting Se concentration in grains **(a)**, straws **(b)**, and roots **(c)** of highland barley. ** indicates *p* < 0.01; * indicates *p* < 0.05; ns indicates *p* > 0.05.

Soil Se dynamics revealed significant treatment effects, with mobility factors (MF) increasing from 0.26 (CK) to 0.58 (100 g·ha^-1^SeVI) (**Supplementary Table S1**), reflecting enhanced bioavailability of Se-amended soil. Strong correlations emerged between grain Se and SOL-Se (*r*=0.82, *P*<0.01), EXC-Se (*r* = 0.76, *p* < 0.01), and MF (*r* = 0.69, *p* < 0.01) (**Figure 6**). SOL-Se explained 14.7% of grain Se variance (%IncMSE = 14.7), surpassing *STSe* (9.3%), indicating the critical role of dissolved Se in root uptake processes. Root Se showed independence from *STSe* (*p* = 0.12) but strong MF dependence (%IncMSE = 18.3), and the machine learning feature importance analysis (**Figure 7**) identified SOL-Se and FMO-Se as primary drivers (combined %IncMSE > 30%).

### 3.4 Assessment of dietary Se intakes from Se-enriched highland barley grain

**Figure 8:**
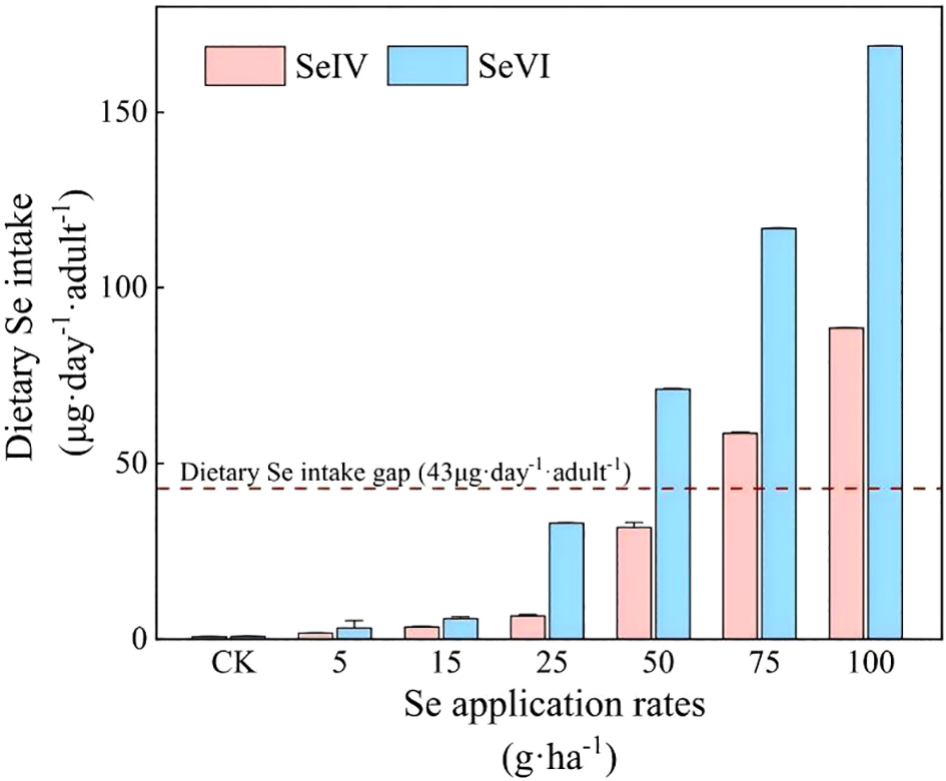
Contribution of Se-enriched highland barley to dietary Se intake of residents (The dashed line shows the gap of dietary Se intake: 43 µg·adult^-1^·day^-1^).

According to the Chinese Nutrition Society’s recommended dietary Se intake for adults (60 ~ 400 µg·adult^-1^·day^-1^) (Chinese Nutrition Society, 2013), and in light of our previous survey results showing that the current dietary Se intake among adult residents in the study area is only 17 µg·adult^-1^·day^-1^ (Zhou et al., 2022), there is a significant deficit of 43 µg·adult^-1^·day^-1^ in dietary Se intake for adult residents in agricultural areas of Tibet. As illustrated in **Figure 8**, the soil application of 75 and 100 g·ha^-1^ SeIV and 50, 75, and 100 g·ha^-1^ SeVI resulted in Se-enriched highland barley grains capable of effectively bridging the dietary Se intake gap of 43 µg·adult^-1^·day^-1^. The Se-enriched highland barley grains produced under these treatments could elevate the total dietary Se intake to the recommended level of 60 µg·adult^-1^·day^-1^, while ensuring that the dietary Se intake remained below the upper threshold of 400 µg·adult^-1^·day^-1^ (**Table 1**). Notably, the dietary Se intake derived from highland barley treated with 75 and 100 g·ha^-1^ of SeIV accounted for 107.52% and 147.53% of the recommended intake value, respectively, while constituting only 14.64% and 22.13% of the upper intake threshold (**Table 1**). For highland barley treated with 50, 75, and 100 g·ha^-1^ SeVI, the dietary Se intake corresponded to 118.38%, 194.63%, and 281.19% of the recommended intake value, while accounting for only 17.75%, 29.20%, and 42.18% of the upper intake thresholds, respectively (**Table 1**). Therefore, we conclude that the soil application of 50 g·ha^-1^ SeVI and 75 g·ha^-1^ SeIV represents the optimal treatments for producing Se-enriched highland barley in Tibet, as these levels ensure adequate Se enrichment without exceeding safe dietary limits.

**Table 1:** Proportion of dietary Se intake of residents (DI) to the recommended amount (RDI) and upper intake threshold (UL).

Se rates (g·ha^-1^)	SeIV		SeVI	
	DI/RDI (%)	DI/UL (%)	DI/RDI (%)	DI/UL (%)
CK	1.20 ± 0.10Ag	0.18 ± 0.02Ag	1.20 ± 0.10Ag	0.18 ± 0.02Ag
5	2.93 ± 0.01Bf	0.44 ± 0.01Bf	5.09 ± 0.36Af	0.76 ± 0.06Af
15	5.72 ± 0.04Be	0.86 ± 0.01Be	9.55 ± 0.10Ae	1.43 ± 0.02Ae
25	11.01 ± 0.08Bd	1.65 ± 0.01Bd	54.83 ± 0.04Ad	8.23 ± 0.01Ad
50	52.89 ± 0.25Bc	7.93 ± 0.04Bc	118.38 ± 0.05Ac	17.76 ± 0.01Ac
75	107.58 ± 0.08Bb	14.64 ± 0.01Bb	194.64 ± 0.03Ab	29.19 ± 0.01Ab
100	147.53 ± 0.03Ba	22.13 ± 0.01Ba	281.19 ± 0.02Aa	42.18 ± 0.01Aa

## 4 Discussion

A strong linear dose-response relationship (*R*² = 0.83~0.94, *p* < 0.001) was observed between Se application rates and tissue-specific accumulation patterns in highland barley (**Figure 2**), validating soil-applied Se as a reliable biofortification strategy for Tibetan plateau agroecosystems. These results corroborate established Se biofortification mechanisms in wheat (*R*² = 0.79~0.91) and rice, confirming conserved Se assimilation pathways in Poaceae species (Ramkissoon et al., 2019; Radawiec et al., 2021; Lessa et al., 2019). Our results further showed that SeIV demonstrated 89% greater grain enrichment efficiency than SeVI (*p* < 0.001), attributable to its rapid conversion to SeMet in rhizospheric redox conditions (Liu et al., 2021). At maximum application (100 g·ha^-1^), SeIV-applied soils produced 1.9 fold higher grain Se concentrations than SeVI equivalents (95% CI: 1.82~2.01; **Supplementary Figure S1**), consistent with phytoavailability patterns in alkaline soils (Liu et al., 2021). The difference mechanism between the efficient enrichment of SeIV in barley grains (68% organic Se) and the wheat system might stem from the synergistic effect of the high pH value (7.2) and calcium carbonate content (18.79 g·kg^-1^) in the soil of the Qinghai-Tibet Plateau. These physicochemical properties enhance the conversion efficiency of SeIV to the organic form by influencing the adsorption-desorption equilibrium of SeIV.

Field trials with wheat demonstrated 590% and 260% Se accumulation enhancement under SeVI and SeIV treatments respectively at 14 g·ha^-1^, highlighting species-dependent assimilation efficiency (Ros et al., 2016). This divergence originates from differential Se speciation: SeVI primarily exists as mobile SeO_4_^2-^ anions, while SeIV forms surface-adsorbed SeO_3_^2-^ complexes with iron oxides (Poblaciones et al., 2024). SeVI utilizes sulfate transporters (Sultr1;1 and Sultr1;2) for xylem loading (Wang et al., 2022), achieving 82% translocation efficiency to aerial tissues versus 37% for SeIV, as quantified by stable isotope tracing (Sors et al., 2005; Poblaciones et al., 2024). SeIV undergoes rapid enzymatic reduction to Se^0^ via glutathione reductase (GR), subsequently incorporating into SeCys2 and SeMet through cysteine synthase pathways, with 68% retention in root tissues as quantified by μ-XANES analysis (Li et al., 2008; Liu et al., 2021).

This study demonstrated that both the application rate and the form of exogenous Se significantly influenced the content and speciation of organic Se in highland barley grains. Notably, organic Se fractions exhibited a dose-dependent decline with increasing SeVI application, inversely correlating with SeIV-treated systems (**Figure 3**). These contrasted with SeVI-fertilized wheat systems where organic Se increased 18~32% with application rates up to 50 g·ha^-1^, attributable to enhanced xylem transport efficiency (Zhang et al., 2018). Meanwhile, In an experimental study by Gong et al. (2018), SeIV-amended soils induced sequential depletion of gluten-bound Se (23~15%) and SeMet (65~48%) in developing caryopses, concomitant with SeCys2 accumulation (8~22%) during grain filling stages. The reasons for the differences may be related to crop types and the specificity of genotypes. Wheat is a moderately Se-accumulating crop, and its sulfur metabolism pathway has a strong ability to integrate Se; while highland barley may have a lower absorption and transformation efficiency of SeVI due to genotype limitations (such as lower activity of sulfur transport proteins), resulting in a decrease in organic Se content with increasing application rate. In wheat grains, glutenin may preferentially bind to SeMet, while in highland barley, due to structural differences in glutenin or fewer Se binding sites, Se is more likely to exist in a free state, such as SeCys2 (Poblaciones et al., 2024). This study also revealed that, following the application of equal concentrations of SeIV and SeVI to the soil, the content of organic Se speciations in highland barley grains was significantly higher in the SeIV treatment compared to the SeVI treatment. Lavu et al. (2012) demonstrated that soil application of SeIV resulted in 79% of organic Se speciation in *Allium tuberosum*, contrasting with 45% observed under SeVI treatment. Isomolar application demonstrated 89% greater grain Se biofortification efficiency under SeIV versus SeVI treatment (*p* < 0.001), with SeMet/SeCys2 ratios diverging significantly. Synchrotron-based μ-XANES analysis confirmed 68 ± 5% selenite(IV) conversion to SeMet/SeCys2 in rhizospheric microzones within 24 h, mediated by glutathione reductase (GR) and cysteine synthase complexes (Nothstein et al., 2016). Our findings corroborate these mechanisms, revealing that SeIV amendment significantly enhanced SeCys2 biosynthesis, with subsequent phloem-mediated allocation to highland barley grains.

Our findings demonstrate that SeIV and SeVI soil applications differentially modified total Se content (*STSe*) and speciation hierarchy, resulting in distinct bioavailability patterns in highland barley cultivation systems (*p* < 0.001). SeIV application (5~100 g·ha^-1^) induced 3.16-76.80-fold *STSe* enhancement, exhibiting significantly stronger soil retention than SeVI (1.65~15.47-fold), due to phosphate-mediated ligand exchange in alkaline soils. However, plant uptake and accumulation of Se depended more on the abundance of available Se than on *STSe* (Wang et al., 2012; Qin et al., 2012). Machine learning feature importance analysis (%IncMSE) revealed bioavailable Se (SOL-Se) accounted for 14.7% of grain accumulation variance, surpassing *STSe* (9.3%)(**Figure 7**), consistent with phytoavailability models in cereal crops (Wang et al., 2013b). In this study, while *STSe* exhibited a positive correlation with the Se concentration in grains and straws, the relative importance ranked lower (**Figures 6**, **7**). Furthermore, Se concentration in both grains and straws were all positively correlated with the content of SOL-Se (**Figures 6**, **7**). This suggests that available Se in the soil was more conducive to plant uptake and accumulation of Se by plants compared to *STSe*. Untreated controls exhibited 83.7% of total Se sequestered in stable oxidized forms (FMO-Se: 58.2 ± 3.1%; OM-Se: 25.5 ± 2.4%) (**Figure 5**), aligning with Se speciation patterns in alkaline soils reported by Chen et al. (2010). SeIV application induced EXC-Se dominance (42.7~58.3%), reflecting phosphate-mediated ligand exchange, while SeVI treatment enhanced SOL-Se proportions (36.1~48.2%) through competitive anion adsorption (**Figure 5**). The treatments generated 2.1~3.8-fold increases in bioavailable Se (EXC+SOL-Se), confirming enhanced mobility (MF=0.58) through redox-driven speciation shifts, as documented in paddy systems (Kamei-Ishikawa et al., 2007; Su and Suarez, 2000). SeVI-amended soils exhibited 15.8~28.4% higher available Se than SeIV-treated counterparts (10.5~18.6%, *P*<0.01), attributable to weaker iron oxide adsorption of SeO_4_^2-^ *vs* SeO_3_^2-^ (Peng et al., 2017; Li et al., 2015; Longchamp et al., 2015). SeVI application induced dose-dependent RES-Se accumulation (*R*²= 0.82, *p* < 0.001), with RES-Se proportions increasing from 13.2% to 28.4% across 5~100 g·ha^-1^ SeVI treatments (**Figure 5**). This aligns with Chen et al. (2010) who documented 13~22% RES-Se enrichment in paddy soils under SeVI treatment, particularly noting 18.7% higher RES-Se in rhizosphere versus bulk soil (*p* < 0.05). Parallel studies in Nicotiana tabacum systems demonstrated 15~28% RES-Se accumulation under SeIV treatment (50~200 g·ha^-1^) (Fan et al., 2015). The observed increase in RES-Se content could potentially be attributed to the incorporation of Se into the soil lattice (Lu et al., 2005) or its adsorption via electrostatic interactions with layered double hydroxides or anionic clays (Rovira et al., 2007). Meanwhile, this study also revealed that the strong correlation between the application amounts of SeVI and RES-Se was highly consistent with the adsorption mechanism reported in rice system studies (Fan et al., 2014). This consistency may be attributed to the high cation exchange capacity (28.4 cmol·kg^-1^) and free iron content (12.7 g·kg^-1^) of the tested soil.

Epidemiological studies demonstrate plasma selenium levels of 70~106 µg·L^-1^ correlate with 58~63% reduction in prostate and lung cancer incidence through enhanced glutathione peroxidase activity (Ullah et al., 2019). Se deficiency can lead to a range of health issues, including growth retardation, thyroid dysfunction, and infertility (Yin et al., 2019). Chronic Se overdose (>400 µg·day^-1^) triggers selenosis characterized by hair loss, dermatitis, and, in extreme cases, fatalities (Fairweather-Tait et al., 2011). Therefore, maintaining an appropriate daily Se intake is essential for health. In the control treatment of this study, the dietary Se intake provided by highland barley grains accounted for only 1.2% of the recommended value of 60 µg·adult^-1^·day^-1^, which was insufficient to meet the nutritional needs of the local Tibetan adults. However, following soil application of exogenous Se, highland barley grains enriched with low Se application rate (<50 g·ha^-1^) were insufficient to provide adequate dietary Se (**Figure 8**). Optimal biofortification achieved at 75 g·ha^-1^ SeIV and 50 g·ha^-1^ SeVI, filling the dietary Se intake gap of 43 µg·adult^-1^·day^-1^ for local residents, without exceeding the upper threshold of dietary Se intake (400 µg·adult^-1^·day^-1^) (**Table 1**).

## 5 Conclusion

Exogenous Se application through soil amendment significantly enhanced Se accumulation in all highland barley tissues. SeVI demonstrated superior translocation efficiency across all application rates (5~100 g·ha^-1^), achieving 1.7~2.5 times higher tissue Se concentrations than SeIV (*p* < 0.05). Conversely, SeIV treatments yielded higher organic Se conversion rates, with SeMet constituting 60~62% of speciated Se in grains. Soil Se pools showed dose-dependent enrichment, both *STSe* and bioavailable Se fractions (SOL+EXC-Se) rose after Se soil application. The distribution and behavior of available Se fractions in soil had a substantial impact on the Se concentration in highland barley. Based on the assessment of dietary Se intake, soil application with 75 g·ha^-1^ of SeIV or 50 g·ha^-1^ of SeVI on highland barley was proved to be an effective and safe measure to fill the dietary Se intake gap of local Tibetan adults in rural agricultural areas in Tibet.

## Data Availability

The original contributions presented in the study are included in the article/**Supplementary Material**. Further inquiries can be directed to the corresponding author.
