# Supplementary material for: Impact of soil application with selenite and selenate on ‘soil-highland barley-dietary’ system in Tibet
Source: Front Plant Sci. 2025 Jun 18;16:1589810. doi: 10.3389/fpls.2025.1589810 (PMC12213702; doi:10.3389/fpls.2025.1589810)
Supplement: Supplementary file 1 [file DataSheet1.docx]

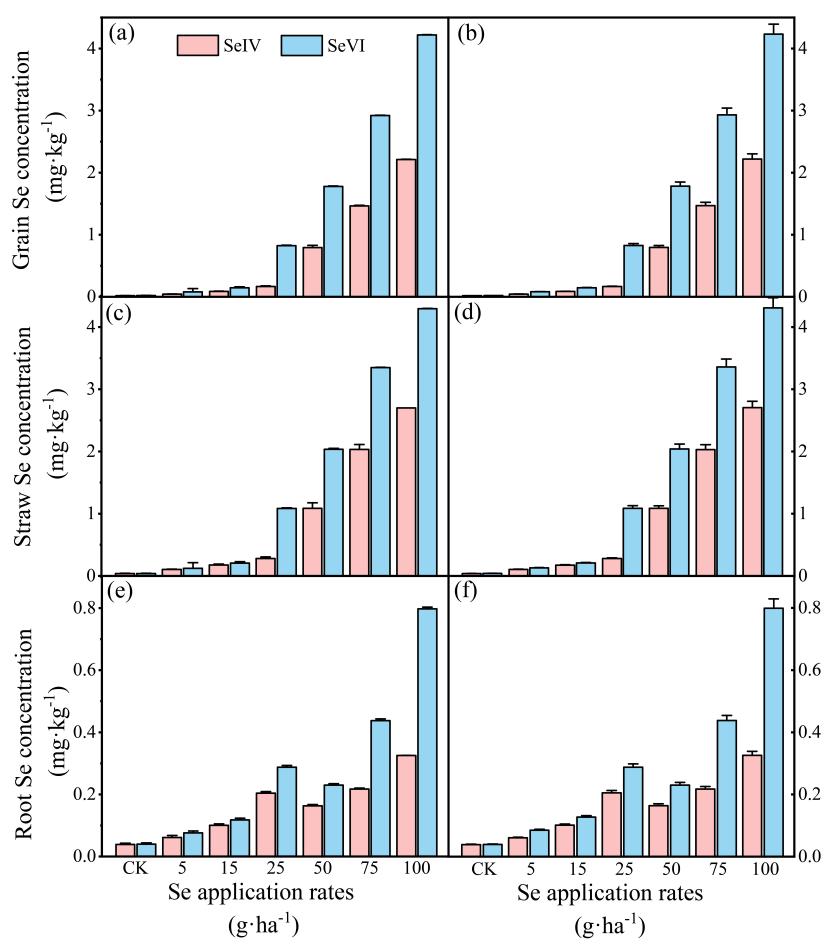


FIGURE S 1 Se concentrations in grain, straw, root of highland barley under different exogenous Se application treatments in 2021 (a,c,e) and 2022 (b,d,f)

TABLE S 1 Parameters characterizing Se behavior in soil

| Se application rates  (g·ha^-1^) | *MF* | | *U_ts_* | | *I_R_* | |
| --- | --- | --- | --- | --- | --- | --- |
|  | SeⅣ | SeⅥ | SeⅣ | SeⅥ | SeⅣ | SeⅥ |
| CK | 26±0.12g | 26±0.12g | 1.00±0.00g | 1.00±0.000g | 0.50±0.01a | 0.50±0.01a |
| 5 | 37±0.26Bf | 43±0.32Af | 1.20±0.02Bf | 1.57±0.02Af | 0.49±0.01Ab | 0.38±0.01Bb |
| 15 | 40±0.38Be | 47±0.24Ae | 1.25±0.01Be | 1.66±0.02Ae | 0.48±0.01Ac | 0.36±0.01Bc |
| 25 | 42±0.26Bd | 50±0.59Ad | 1.29±0.03Bd | 1.72±0.03Ad | 0.47±0.02Ad | 0.34±0.01Bd |
| 50 | 45±0.37Bc | 54±0.42Ac | 1.35±0.02Bc | 1.86±0.02Ac | 0.46±0.02Ae | 0.33±0.01Be |
| 75 | 53±0.48Bb | 60±0.35Ab | 1.60±0.03Bb | 2.07±0.02Ab | 0.41±0.02Af | 0.30±0.01Bf |
| 100 | 60±0.18Ba | 65±0.30Aa | 1.88±0.02Ba | 2.30±0.01Aa | 0.37±0.02Ag | 0.27±0.01Bg |
| Se application rate (A) | *P*＜0.001 | *P*＜0.001 | *P*＜0.001 | *P*＜0.001 | *P*＜0.001 | *P*＜0.001 |
| Exogenous Se type (B) | *P*＜0.001 | *P*＜0.001 | *P*＜0.001 | *P*＜0.001 | *P*＜0.001 | *P*＜0.001 |
| A×B | *P*＜0.001 | *P*＜0.001 | *P*＜0.001 | *P*＜0.001 | *P*＜0.001 | *P*＜0.001 |

Note: Values are means of four replicates. For each cropping season, means in a column followed by different capital letters on behalf of significant differences among two exogenous Se types under the same Se application rate; means in a column followed by different lowercase letters on behalf of significant differences among 7 Se application rates under the same exogenous Se types
